# D:L-Amino Acid Modeling Reveals Fast Microbial Turnover of Days to Months in the Subsurface Hydrothermal Sediment of Guaymas Basin

**DOI:** 10.3389/fmicb.2018.00967

**Published:** 2018-05-15

**Authors:** Mikkel H. Møller, Clemens Glombitza, Mark A. Lever, Longhui Deng, Yuki Morono, Fumio Inagaki, Mechthild Doll, Chin-chia Su, Bente A. Lomstein

**Affiliations:** ^1^Center for Geomicrobiology, Department of Bioscience, Aarhus University, Aarhus, Denmark; ^2^Section for Microbiology, Department of Bioscience, Aarhus University, Aarhus, Denmark; ^3^National Aeronautics and Space Administration-Ames Research Center, Moffett Field, CA, United States; ^4^Department of Environmental Systems Science, Institute of Biogeochemistry and Pollutant Dynamics, ETH Zürich, Zürich, Switzerland; ^5^Geomicrobiology Group, Kochi Institute for Core Sample Research, Japan Agency for Marine-Earth Science and Technology, Yokosuka, Japan; ^6^Faculty of Geosciences (FB 05), University of Bremen, Bremen, Germany; ^7^Department of Mechanical Engineering, National Taiwan University, Taipei, Taiwan

**Keywords:** D:L-model, turnover time, organic matter quality, degradation, amino acid, volatile fatty acid, hydrothermal, Guaymas Basin

## Abstract

We investigated the impact of temperature on the microbial turnover of organic matter (OM) in a hydrothermal vent system in Guaymas Basin, by calculating microbial bio- and necromass turnover times based on the culture-independent D:L-amino acid model. Sediments were recovered from two stations near hydrothermal mounds (<74°C) and from one cold station (<9°C). Cell abundance at the two hydrothermal stations dropped from 10^8^ to 10^6^ cells cm^-3^ within ∼5 m of sediment depth resulting in a 100-fold lower cell number at this depth than at the cold site where numbers remained constant at 10^8^ cells cm^-3^ throughout the recovered sediment. There were strong indications that the drop in cell abundance was controlled by decreasing OM quality. The quality of the sedimentary OM was determined by the diagenetic indicators %T_AA_C (percentage of total organic carbon present as amino acid carbon), %T_AA_N (percentage of total nitrogen present as amino acid nitrogen), aspartic acid:β-alanine ratios, and glutamic acid:γ-amino butyric acid ratios. All parameters indicated that the OM became progressively degraded with increasing sediment depth, and the OM in the hydrothermal sediment was more degraded than in the uniformly cold sediment. Nonetheless, the small community of microorganisms in the hydrothermal sediment demonstrated short turnover times. The modeled turnover times of microbial bio- and necromass in the hydrothermal sediments were notably faster (biomass: days to months; necromass: up to a few hundred years) than in the cold sediments (biomass: tens of years; necromass: thousands of years), suggesting that temperature has a significant influence on the microbial turnover rates. We suggest that short biomass turnover times are necessary for maintance of essential cell funtions and to overcome potential damage caused by the increased temperature.The reduced OM quality at the hyrothemal sites might thus only allow for a small population size of microorganisms.

## Introduction

Microorganisms buried in the seabed play a crucial role in the degradation of organic matter (OM) in the sediments. A substantial part of the Earth’s prokaryotic cells (∼3 × 10^29^ according to Kallmeyer et al., 2012) exist below the seafloor where they cope with many challenges such as increasing pressure, low energy and nutrient availability and increasing temperatures. Although extensively studied during the past three decades, many details about the workings of life in the deeply buried environments remain unclear. Guaymas Basin offers a unique opportunity to study deeply buried organisms in high temperature environments because the active seafloor spreading in this marginal rift basin co-occurs with the rapid deposition of OM rich sediments from the overlaying highly productive waters (Calvert, 1966). Spreading centers are found globally on the ocean floor (Dick et al., 2013) but many of these are located in the deep oceans and thus are characterized by low rates of OM deposition from overlying water or from land and consequently low sedimentation rates. However, in Guaymas Baisn, the high productivity of the water column together with significant terrestrial sediment input result in a several hundred meter thick sediment cover (Einsele et al., 1980). During seafloor spreading, basaltic magma intrudes as sills into the sediment, heating the pore water and creating hydrothermal fluids that percolate through the thick OM rich sediment and expose the sediment to temperatures of >300°C (Einsele et al., 1980; Lonsdale and Becker, 1985; Von Damm et al., 1985).

The ratio between the two amino sugars glucosamine (GlcN) and galactosamine (GalN) (Benner and Kaiser, 2003; Niggemann and Schubert, 2006) as well as the ratio between the two amino acids glycine (Gly) and serine (Ser) (Keil et al., 2000) are well proven OM source indicators. These ratios differ between prokaryotes and eukaryotic organisms such as phytoplankton and zooplankton, and can provide an indication of the predominant source of the OM deposited and present in the sediment. Proteins are a vital component of all living organisms build by combining different amino acids into larger functional macromolecules. The contribution of amino acid carbon and amino acid nitrogen to total organic carbon (TOC) and total nitrogen has previously been used to evaluate the degradation status of OM (Cowie and Hedges, 1994; Lomstein et al., 2006). Likewise, the ratio between specific amino acids and their non-protein-bound degradation products can be used as indicator of the diagenetic state of sedimentary OM (Cowie and Hedges, 1994; Dauwe and Middelburg, 1998; Langerhuus et al., 2012), where the quantity of degradation products increases relative to the quantity of precursor amino acids as OM becomes increasingly degraded.

Microorganisms inhabiting sediments below the photic zone gain most of their energy by metabolizing organic compounds that originate from the photosynthetic biosphere and dead microbial biomass (necromass). Sediment microbial activity is thus highly dependent on the amount and quality of OM that is accessible. Previous studies have shown that microorganisms in the deep biosphere metabolize at extremely low rates compared to surface environments, and that the activity becomes gradually lower with depth as the energy sources become scarce (Lever et al., 2015a; Jørgensen and Marshall, 2016). Microbial activity in marine sediments has been widely measured by laboratory incubation experiments using ^35^S-labeled sulfate turnover to measure sulfate reduction rates (Jørgensen and Fenchel, 1974; Jørgensen, 1977, 1982). As an alternative to the radiotracer experiments, Lomstein et al. (2012) published a model that is culture-independent to quantify the turnover times of microbial necromass and living microbial biomass. This D:L-amino acid model is based on the build-in molecular clock of the amino acid aspartic acid (Asp), i.e., the chemical racemization that randomly converts the conformation of the amino acid between the D- and L-isomer form. By applying this model, a recent study from Braun et al. (2017) showed microbial biomass turnover times of months to decades (maximum up to ∼120 years) in a variety of Holocene and Pleistocene marine sediments, with temperatures <20°C. The authors suggested that the relatively short turnover time reflects microbial metabolic and physiologic adaptations to the energy deprived deep biosphere, such as extremely low cell-specific energy demand, a small cell body size and an enhanced ability to degrade amino acids and total uncharacterized organic matter in stationary phase.

In the current study, we applied the D:L-amino acid model for the first time to a high temperature environment, the hydrothermal system of Guaymas Basin. The selected study sites were characterized by high *in situ* temperatures (up to 74°C) falling within the known temperature regime of life (Takai et al., 2008) in combination with a low temperature reference site (<9°C). We estimated microbial activity from biomass and necromass turnover times by using the D:L amino acid model. In order to evaluate the controls on microbial activity in these hydrothermal sediments, we evaluated the quality and origin of the organic matter in a comprehensive analysis of the concentration and composition of amino acids and volatile fatty acids (VFAs).

## Materials and Methods

### Study Site and Sampling

The sediment was collected during the R/V *Sonne* expedition 241 in June–July, 2015. At Stations SO241-51 and SO241-58, two gravity cores were recovered from an area located in close proximity to a hydrothermal vent site in the southern part of the northern trough of Guaymas Basin at a water depth of ∼1800 m (**Table 1**; Berndt et al., 2015). *In situ* temperatures reach up to 74 and 66°C in the deepest part of Station 51 and Station 58, respectively. The sediment at the base of the two gravity cores consists of black, fine-grained, metal-rich sediment overlain by hemipelagic sediment with intercalated hydrothermal deposits (Berndt et al., 2015). A third gravity core was retrieved at Station SO241-46 outside the hydrothermal vent field (<9°C). This station is located at a water depth of 664 m in the oxygen minimum zone (OMZ), which extends here all the way to the seafloor. The sediments at this site are characterized by high accumulation and burial rates of labile, phytoplankton-derived organic matter and are further influenced by minimal terrestrial OM input from the Yaqui River.

**Table 1:** Sampling sites including the geographic position of the stations, water depth, core recovery, sedimentation rate, temperature gradient, maximum *in situ* temperature, and type of location.

Station	Geographic coordinates^a^	Water depth^a^ (m)	Core recovery^a^ (cm)	Sedimentation rate (mm yr^-^^1^)	Temperature in deepest sample^b^ (°C)	Temperature gradient^b^ (°C/m)	Location type^a^
SO241-46	27° 42.412′N 111° 13.651′W	664	1000	2.10	8.83	0.13	Marine, with minimal terrestrial influence, OMZ
SO241-51	27° 24.472′N 111° 23.377′W	1840	487	0.79	74.27	11.44	Hydrothermal
SO241-58	27° 24.487′N 111° 23.377′W	1845	498	0.79	65.51	9.86	Hydrothermal

^a^*Berndt et al. (2015); ^*b*^Temperatures were measured with miniaturized temperature data loggers (Pfender and Villinger, 2002)*.

Immediately after core recovery, 10 samples were taken from each core with 5 mL cut-off syringes and stored at -20°C (Berndt et al., 2015) for later analysis of amino acids, amino sugars, TOC and total nitrogen in the home laboratory. The sample intervals at Station 51 and 58 were 50 cm starting from 37 and 48 cm below seafloor (cmbsf), respectively. The core from Station 46 was sampled every 100 cm starting at 250 cmbsf (Berndt et al., 2015).

Sediment samples for DNA extraction and cell extraction were taken using sterile, cut-off 3-cc syringes. Samples for DNA extraction were immediately frozen at -80°C after sampling, whereas samples for cell enumeration were immediately fixed (see section “Total Cell Counts”). Samples for radionuclide analyses were taken by sectioning multi-cores along distinct horizons with a decrease in the depth resolution as sediment depth increased (0–4 cmbsf: 1-cm depth intervals; 4–20 cmbsf: 2-cm depth intervals; 20–40 cmbsf: 4-cm depth intervals).

Pore water for VFA analysis was sampled from the cores in a resolution of 50 cm (Station 51 and 58) or 1 m (Station 46). Approximately 100 cm^3^ of sediment were sampled into several acid washed 50-cm^3^ falcon tubes and centrifuged in a cooled centrifuge for 30–60 min. The supernatant was collected in baked (5 h at 450°C) 4-mL glass vials (Zinsser Analytic GmbH, Germany), frozen at -80°C and shipped to Aarhus University (Denmark) for analysis.

### Total Cell Counts

Total cell counts were performed by cell fixation, followed by cell detachment, and quantification by flow cytometry (FCM). Cell fixation was done according to Langerhuus et al. (2012). 0.5 cm^3^ fresh sediment aliquots were homogenized with 0.5 mL of cell fixation solution (4% paraformaldehyde [PFA], 3% sodium chloride) and incubated for 2 h or overnight at 4°C. PFA was then removed by washing twice with 1 mL phosphate buffered saline (PBS) followed by centrifugation for 10 min at 10,000 × *g* and removal of supernatants. The final sediment pellet was resuspended in 0.5 mL PBS and 0.75 mL 96% ethanol (EtOH), and stored at -20°C.

Cell detachment was done as outlined in Morono et al. (2009). 600 μl of 2.5% NaCl solution, 100 μl of detergent mix (100 mM EDTA, 100 mM sodium pyrophosphate, 1% (v/v) Tween 80), and 100 μl methanol were added to 100 μl diluted sediment slurry (1:20 to original sample, diluted with 2.5% NaCl solution). Next, samples were vigorously shaken for 60 min at 500 rpm using a Shake Master (Biomedical Science, Tokyo, Japan). After shaking, the sediment slurry was sonicated at 160 W for 20 min using a Bioruptor UCD-250 Sonicator (Cosmo Bio Co., Ltd., Tokyo, Japan). 100 μl of 10% [wt/v] hydrofluoric acid (HF) was added and incubated for 20 min at room temperature. The reaction was stopped by addition of stop solution (1 M Tris–hydrochloric acid [HCl, pH 8.0], 0.125M CaCl2 and 25% methanol).

Lastly, the cells were analyzed by flow cytometry (FCM) according to Morono et al. (2013). 10 μl of the sediment suspension in 1000 μl of TE buffer was placed onto an Anopore Inorganic Membrane (Anodisc, Whatman, Kent, United Kingdom), washed with TE buffer and then stained with 40 μl of SYBR Green I (Molecular Probes-Invitrogen, Carlsbad, CA, United States) staining solution (1/40 [v/v]). After staining for 5 min, the SYBR-stained cells were washed with 2 mL of TE buffer, and then the membrane was placed into a 15 mL centrifuge tube containing 2 mL of TE buffer. Cells were detached from the membrane by sonication at 160 W for 1 min using a Bioruptor UCD-250 Sonicator (Cosmo Bio Co., Ltd., Tokyo, Japan). The cell suspension was then transferred to Falcon^TM^ Tube with Cell Strainer Cap (Mesh size: 35 μm, Corning, NY, United States). For volumetric calibration, custom-made fluorescent beads (Green [505/515 nm] and Deep Red [633/660 nm] double color) were added at a concentration of 4.5 × 10^4^ beads cm^-3^. The cell suspension was analyzed using a Gallios flow cytometer (Beckman Coulter), and the FCM data were analyzed using Kaluza analysis software (Beckman Coulter).

### Bacteria and Archaea Quantification

DNA was extracted using lysis protocol II (LP II) as described in Lever et al. (2015b). Sediment samples (∼0.2 g) were thawed and placed in 2-mL screw-cap tubes containing ∼15% v/v 0.1-mm zirconium silica beads, homogenized with 100 μL of 10 mM sodium hexametaphosphate solution by gentle shaking, and re-frozen. Samples were then thawed and amended with 500 μL lysis solution I, homogenized for 30 s on a Qiagen TissueLyzer set to 30 shakings s^-1^, and incubated for 1 h at 50°C on an Eppendorf ThermoMixer Comfort set to 600 rpm. After this treatment, DNA extracts were separated from sediments by centrifugation at 10,000 × *g* for 20 mins at 4°C, washed twice with ice-cold chloroform-isoamylalcohol (24:1 v:v), and precipitated using ethanol-sodium chloride after addition of linear polyacrylamide (LPA) of extract before ethanol-sodium chloride addition. After 2 h of incubation at room temperature, the samples were centrifuged at 14,000 × *g* for 30 min. The supernatant was carefully pipetted off or decanted, and the residue containing the nucleic acids was air-dried. The dry residue pellet was dissolved in 100 μL of water and purified using a Norgen CleanAll RNA/DNA Clean-Up and Concentration Kit. After this final treatment the samples were ready for downstream quantifications and qPCR.

Bacterial and archaeal 16S rRNA gene copy numbers were quantified by quantitative real-time PCR (qPCR), on a PikoReal Real-Time PCR System (Thermo Fisher Scientific) using 5 μL of the Roche LightCycler 480 SybrGreen I mixture, 1 μL of each 10 μM primer solution, 1 μL of 1 mg mL^-1^ bovine serum albumin, and 2 μL DNA/cDNA template solution. The primer pairs used in separate assays were Bac908F_mod (Lever et al., 2015b) and Bac1075R (Ohkuma and Kudo, 1998) for Bacteria quantification, and Arc915Fmod (Cadillo-Quiroz et al., 2006) and Arc1059R (Yu et al., 2005) for Archaea quantification, and followed the protocols outlined in Lever et al. (2015b). Apart from the different primer pairs used, the qPCR protocols were carried out under similar conditions as follows: first, 95°C polymerase activation was carried out for 5 min, followed by 45–50 PCR cycles. The PCR cycles consisted of (1) denaturation for 30 s at 95°C, (2) annealing for 30 s at 60°C or 55°C for Bacteria and Archaea, respectively, (3) elongation for 30 s at 72°C, and (4) fluorescence measurements after 5 s at 80°C. Each qPCR run ended with a stepwise melting curve from 95°C to 55°C for 1 min, to check for primer specificity. The standard curves were based on pGEM-T plasmids (Promega) with archaeal or bacterial 16S rRNA gene inserts. Archaeal and bacterial cell numbers were calculated by assuming 4.0 and 1.6 gene copies per Bacteria and Archaea, respectively. Gene copy numbers were retrieved from “The Ribosomal RNA Operon Copy Number Database” on April 20th, 2016 (Cole et al., 2014^1^).

### Preparation of Samples for Amino Acid and Amino Sugar Analyses

Sediment samples were freeze-dried and homogenized, except for the last cm of sediment in the cut-off syringe, which were disposed. Approximately 0.25 g of freeze-dried sediment was added to an injection flask and 5 mL 6 M HCl was added. The headspace of the injection flask was replaced with N_2_ and the sample was hydrolysed for 24 h at 105°C. Subsequently, the sample was transferred into an ice bath to stop the hydrolysis.

As described by Lomstein et al. (2006), evaporation was carried out to remove volatile compounds by transferring 400 μL hydrolysate into a glass scintillation vial placed in a desiccator, at 45°C over night at reduced pressure. Dried samples were dissolved in 400 μL Milli-Q^®^ water, and evaporated again over night. The dried and evaporated samples were dissolved in 4 mL Milli-Q^®^ water and filtered through Q-Max^®^CA-S Syringe Filters (pore size 0.2 μm) into pico-vials of which the first milliliter was discarded.

### Total Organic Carbon and Total Nitrogen

The concentrations of TOC and total nitrogen (TN) were measured by adding approximately 25–50 mg of freeze-dried sediment sample (depending on the expected concentration of TOC and TN) to small tin cups, after removal of inorganic carbon with 5–6% (w:w) sulfurous acid as described by Braun et al. (2017). Samples were then analyzed in a Flash EA 1112 HT Elemental Analyser (Thermo Scientific) equipped with a TCD (Thermal Conductivity Detector). The TOC and TN concentrations were calculated from an external 5–7 point standard calibration curve, using flour containing 44.39% carbon and 2.31% nitrogen as a standard. Blanks showed negligible amounts of carbon and nitrogen.

### THAA and Amino Sugars

The concentrations of total hydrolysable amino acids (THAAs) and amino sugars (AS) were determined by reverse-phase, gradient and high performance liquid chromatography (HPLC) with fluorescence detection. Prior to analyses, amino acids and amino sugars were derivatized with fluorescent o-phthaldialdehyde (OPA) according to the method of Lindroth and Mopper (1979) with the modifications described by Langerhuus et al. (2012). The Sentry TM guard column was replaced with a Waters Nova-Pak^®^ (4 μm) 3.9 mm × 20 mm guard column. An adequate amount (400–800 μL) of the dissolved samples after evaporation was diluted and transferred to Teflon vials for HPLC-analysis. Blanks were prepared and treated like the samples, with the exception that sediment was omitted. Blanks only showed negligible amounts of amino acids compared to samples.

The two amino sugars galactosamine (GalN) and glucosamine (GlcN) were analyzed by HPLC using the method for THAA-analysis as described above. Every sample was analyzed twice whereby one of the samples was spiked with a 25 nM standard mix of GalN and GlcN in order to ensure correct quantitative determination of the samples. Concentrations were corrected for losses during hydrolysis, which amounted to 25.5% for GlcN and 21.6% for GalN (Lomstein et al., 2009).

### D:L-Amino Acid Isomers

The concentrations of D- and L-isomers of Asp were determined by the HPLC method described by Mopper and Furton (1991) with the modifications given in Langerhuus et al. (2012), including the following additional modifications: (1) N-isobutyryl-D-cysteine (IBDC) was used as a chiral agent added to OPA, (2) the pH of eluent A was adjusted to 6.4, (3) L-glutamine was used as an internal standard, and (4) all samples were analyzed twice, whereby one of the samples was spiked with a 25 nM standard. The concentrations of D- and L-aspartic acid were corrected for racemization during hydrolysis according to Kaiser and Benner (2005). They found that the average percentage of D-enantiomers (% [D/(D+L)]) produced during hydrolysis was 4.4% for Asp.

### Volatile Fatty Acids

VFA concentrations in the pore water samples were measured by two-dimensional ion chromatography mass spectrometry (2D IC-MS; Glombitza et al., 2014). Prior to analyses, the samples were filtered through disposable Acrodisc^®^ 13 mm IC syringe filters (pore size 0.2 μm) that were flushed with 10 mL Milli-Q^®^ water directly before use. The first 0.5 mL of filtered porewater was discarded and a second 0.5 mL was used for analysis. The instrument used for 2D IC-MS analysis was a Dionex ICS3000 coupled to a Surveyor MSQ Plus mass spectrometer (both Thermo Scientific). The method is described in detail in Glombitza et al. (2014). Briefly, in this method the first IC dimension is used to separate inorganic ions, such as chloride, from VFAs. VFAs are trapped on a concentrator column and subsequently separated in the second IC dimension. Quantification is achieved by the mass spectrometer in the single ion-monitoring (SIM) mode. Detection limits for individual VFAs are all between 0.1 and 0.5 μM. Quantification was achieved by a 3-point calibration with external standards of a mixture of VFAs (formate, acetate, propionate) at different concentrations (i.e., 200, 500, and 800 μg L^-1^) in IAPSO seawater standard (OSIL, United Kingdom).

### Sedimentation Rates

The sedimentation rates were determined by the method described by Berndt et al. (2015). The wet sediment was weighed, freeze-dried at -80°C and reweighed to determine the water content and subsequently ground in a mortar. Radionuclides were then measured as follows: two HPGe detectors were engaged for ^210^Pb and ^226^Ra analysis including GMX-type (ORTEC GMX-120265) and well-type (ORTEC GWL-100230) detectors, which interfaced to a digital gamma-ray spectrometer (DSPecPlus^TM^). For the GMX-type detector, absolute counting efficiencies for various photon energies were calibrated using IAEA reference materials 327A, 444 spiked soil, CU-2006-03 spiked soil, RGTh and RGU for sample weight at 100 g as a reference, and coupled with an in-house secondary standard for various masses (from 10 to 250 g) to calibrate the effect of sample mass on the attenuation of γ-ray of various energies. For the well-type detector, the counting efficiencies were calibrated by IAEA-RGTh and RGU from 0.5 to 3.5 g. ^214^Pb was used as an index of ^226^Ra (supported ^210^Pb) whose activity concentration was subtracted from that of the measured total ^210^Pb to obtain excess ^210^Pb (^210^Pbex). The ^210^Pb and ^214^Pb activities were quantified based on photon peaks centered at 46.52 and 351.99 keV, respectively. The activities of radionuclides were decay-corrected to the date of sample collection. All radionuclide data were calculated on salt-free dry weight basis.

### The D:L-Amino Acid Model

The primary output of the D:L-amino acid model is the turnover time of microbial necromass, from which the turnover time of living microbial biomass can be calculated (Lomstein et al., 2012). The advantage of the D:L-amino acid model is that microbial activity can be quantified without the use of incubation experiments, in up to several millions of years old sediment. There are two important assumptions to the model: (1) the subsurface total cell counts are in a quasi-steady state on relevant timescales, and (2) there is only one pool of Asp in the microbial necromass; i.e., we do not differentiate between more or less reactive Asp pools.

The D:L-amino acid model divides sedimentary THAA into two pools: THAA in microbial necromass and THAA in living biomass. The amino acid carbon cycles between these pools. When it can be assumed that there is a quasi-steady state in total cell counts then the amount of necromass degraded must equal the amount of new biomass produced.

The input parameters to the D:L-amino acid model are (1) the measured D:L-Asp ratios in the sediment and in vegetative microbial cells extracted from subseafloor sediment, (2) microbial biomass, (3) amino acid nitrogen, (4) the distribution between Bacteria and Archaea, (5) the total concentration of Asp, and (6) the racemization rate constant for Asp.

Living microorganisms repair or replace amino acids that change configuration due to chemical racemization and thus maintain a low D:L-ratio (0.014) according to Braun et al. (2016), whereas the D:L-ratio of microbial necromass approaches chemical equilibrium over time if the microbial necromass is not renewed. How fast chemical equilibrium is reached depends on the temperature, since the racemization rate constant is highly temperature dependent (Bada, 1982; Steen et al., 2013, 2015).

The turnover times of microbial necromass and biomass were calculated according to the principles given in Lomstein et al. (2012) and with the modifications given in Braun et al. (2017).

## Results

### Distribution of Microbial Cells

**FIGURE 1:**
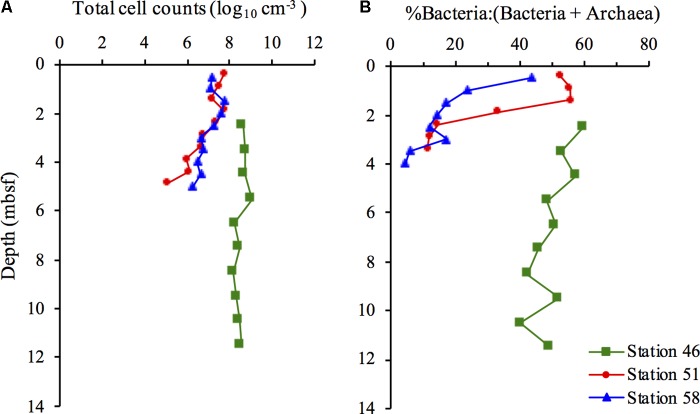
Distribution of total cell counts **(A)** and the relative contribution of Bacteria to (Bacteria + Archaea) **(B)** at Station 46, 51, and 58.

The distribution of total cell counts at the hydrothermal vent Stations 51 and 58 shows distinct differences compared to the cold Station 46. In the upper part of the sediment, at the two hot stations, the total cell counts were in the order of 10^7^–10^8^ cells cm^-3^ after which they decreased with depth to ∼10^6^ at Station 58 and ∼10^5^ cells cm^-3^ at Station 51 at ∼5 mbsf. (**Figure 1A**). At the cold Station 46, the total cell counts remained relatively constant throughout the sediment (∼10^8^ cells cm^-3^) resulting in a 100-fold lower cell abundance at ∼5 mbsf at the hot sites compared to the cold site.

The distribution between Bacteria and Archaea [(% Bacteria:(Bacteria+Archaea)] showed substantial fluctuations at the two hydrothermal vent sites, compared to the cold site (**Figure 1B**). Bacteria and Archaea were nearly equally distributed in the uppermost part of the sediment at Stations 51 and 58, with a gradual decrease in the relative contribution of Bacteria to 5–11% at ∼4 mbsf. The relative contribution of Bacteria gradually decreased from 60% in the upper part of the sediment at Station 46 to 40–49% in the deepest samples.

### Total Organic Carbon and Total Nitrogen

**FIGURE 2:**
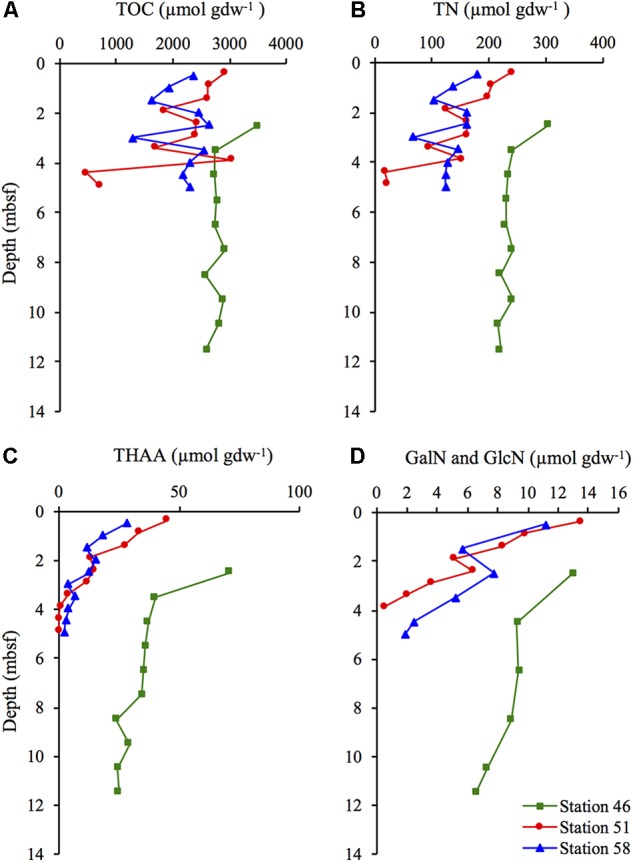
Concentration of **(A)** total organic carbon (TOC), **(B)** total nitrogen (TN), **(C)** total hydrolysable amino acids (THAA) and **(D)** the total concentration of the two amino sugars GalN and GlcN at Station 46, 51, and 58.

The concentrations of TOC and TN did not show any consistent trend with depth at the hydrothermal stations (Station 51 and 58). At Station 46, the concentrations of TOC and TN remained relatively constant at sediment depth >3.5 mbsf (**Figures 2A,B**). At Station 51, the concentrations of TOC varied between 3,030 and 459 μmol gdw^-1^ and between 2,631 and 1,271 μmol gdw^-1^ at Station 58. At Station 51, the TN was in the range of 17–240 μmol gdw^-1^, and in the range of 66–180 μmol gdw^-1^ at Station 58. The TOC and TN concentrations at Station 46 remained relatively constant at sediment depth >2.5 m at ∼2,800 and ∼230 μmol gdw^-1^, respectively.

### Total Hydrolysable Amino Acids and Amino Sugars

The concentration of THAA decreased with depth at all three stations, but the decrease was steepest at the two hydrothermal stations. THAA concentrations decreased from 28 to 2 μmol gdw^-1^ (Station 51) and from 45 to 0.6 μmol gdw^-1^ (Station 58; **Figure 2C**). At Station 46, the concentration of THAA decreased from 71 to 25 μmol gdw^-1^. Concentrations of the two amino sugars (GalN and GlcN) showed a similar decrease with depth as the THAA concentrations. (**Figure 2D**).

### Source Indicators

The ratios between glycine (Gly) and serine (Ser), were relatively stable throughout all three cores, and fell in the range of 2.4–4.1 (data not shown). In prokaryotic biomass the Gly:Ser-ratio is ∼2.3, while the ratio in phytoplankton and diatoms is ∼1.0 and ∼0.7, respectively (Keil et al., 2000). The ratio between GlcN and GalN, fell in the range of 1.2–2.8 at all three stations, with one exception at Station 58 where the GlcN:GalN-ratio was 3.9 at ∼5 mbsf (data not shown).

### Diagenetic Indicators

**FIGURE 3:**
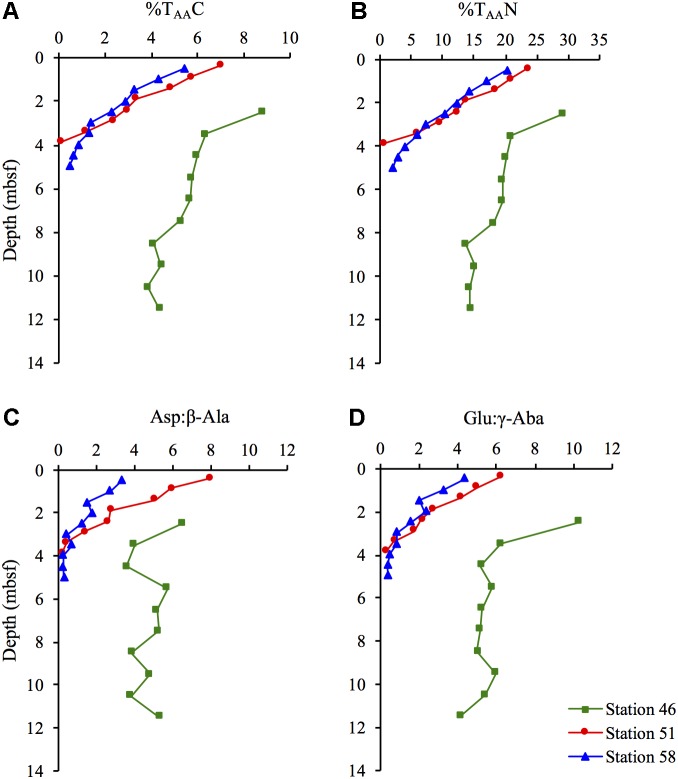
**(A)** percentage of total organic carbon present as amino acid carbon (%T_AA_C) and **(B)** percentage of total nitrogen present as amino acid nitrogen (%T_AA_N) and the ratio between the two amino acid precursors and their degradation products **(C)** Asp:β-Ala, and **(D)** Glu:γ-Aba at Station 46, 51, and 58.

The contribution of amino acid carbon to TOC (%T_AA_C) decreased with depth at all three stations (**Figure 3A**), with a more pronounced decrease at Stations 51 and 58, where the %T_AA_C decreased from 7.0 to 0.1% and 5.5 to 0.5%, respectively. The contribution of amino acid nitrogen to TN (%T_AA_N), showed a similar depth trend as the %T_AA_C decreasing from 24 and 20% in the sediment surface to 0.5 and 2.1% in the deepest part of the of the sediment at Stations 51 and 58, respectively (**Figure 3B**). At Station 46, the %T_AA_N decreased from 29 to 14%.

The ratios between the two amino acids Asp and Glu and their respective non-protein-bound degradation products β-alanine (β-Ala) and γ-amino butyric acid (γ-Aba), showed a similar trend as %T_AA_C and %T_AA_N, with a steep decrease with depth at the hydrothermal stations (**Figures 3C,D**). At Station 46, the two ratios only decreased slightly with increasing sediment depth.

### D-Asp Concentration and D:L-Asp Ratio

**FIGURE 4:**
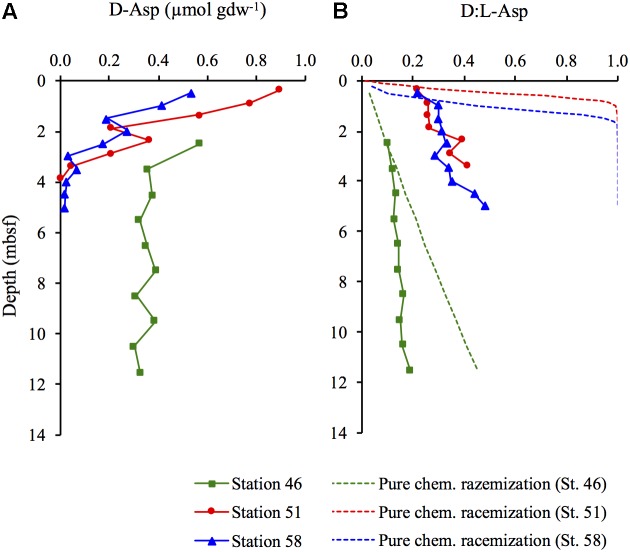
**(A)** Concentration of D-Asp and **(B)** the D:L-Asp ratios including the expected D:L-Asp ratios (dotted lines) from chemical racemization at the *in situ* temperature if there was no new production of microbial necromass.

The concentrations of D-Asp decreased with depth at the two hydrothermal sites (Stations 51 and 58) while they remained constant at depth >3.5 mbsf at the cold site (Station 46) (**Figure 4A**). There was an increase in the D:L-Asp ratio with sediment depth at all three stations (**Figure 4B**). The lowest D:L-Asp ratio (0.101) was measured in the surface of Station 46 and the highest ratio (0.481) was obtained in the deepest part of Station 58. The D:L-Asp ratios in living bacterial cells has been measured in purified sedimentary microbial cell extracts to be 0.014 on average (Braun et al., 2016). The D:L-Asp ratios included in the D:L-amino acid model were all higher than 0.014, and lower than the D:L-Asp that could be predicted from pure chemical racemization at the *in situ* temperature (**Figure 4B**).

**FIGURE 5:**
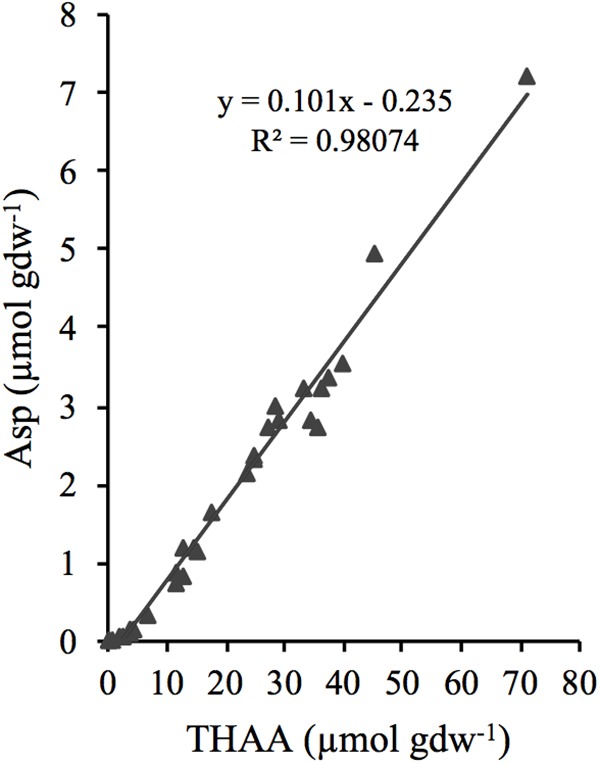
Relationship between the concentration of total hydrolysable amino acids (THAA) and aspartic acid (Asp).

The concentration of Asp was linearly related to THAA (*R*^2^ = 0.98) over the full range of analyzed samples (**Figure 5**), and since the microbial necromass accounted for >99% of the THAA pool, it could be assumed that the D:L-amino acid model does not differentiate between Asp pools of different reactivities.

### D:L-Asp Modeling of Microbial Turnover Times

**FIGURE 6:**
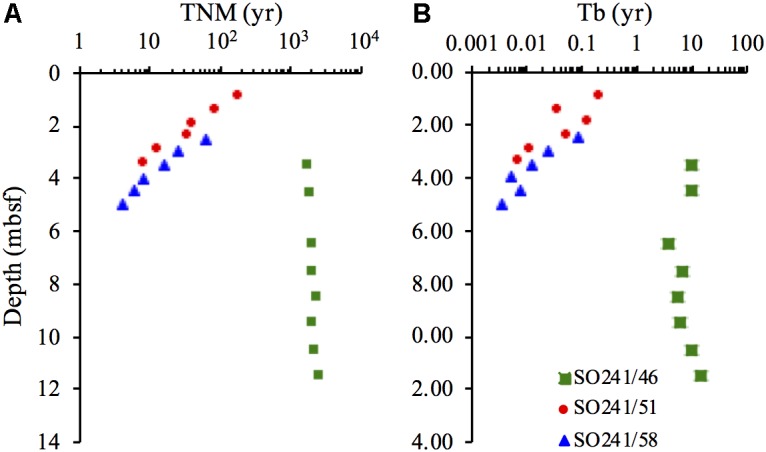
Turnover times of **(A)** living microbial biomass (T_b_) and **(B)** microbial necromass (T_NM_) at Station 46, 51, and 58.

The modeled turnover times of microbial necromass (T_NM_) decreased with depth from 182 to 8 years at Station 51, and from 63 to 4 years ∼5 mbsf, at Station 58 (**Figure 6A**). The T_NM_ at Station 46 increased slightly with depth from 1731 years in the uppermost sample to 2572 years at 11.5 mbsf. The turnover times of microbial biomass (T_b_) followed the same trend as for T_NM_ (**Figure 6B**). At Station 51, T_b_ decreased from 75 to 3 days, while T_b_ decreased from 32 days to 1 day at Station 58. At Station 46, T_b_ was variable and fell in the range of 4–14 years.

### Volatile Fatty Acid Concentrations

**FIGURE 7:**
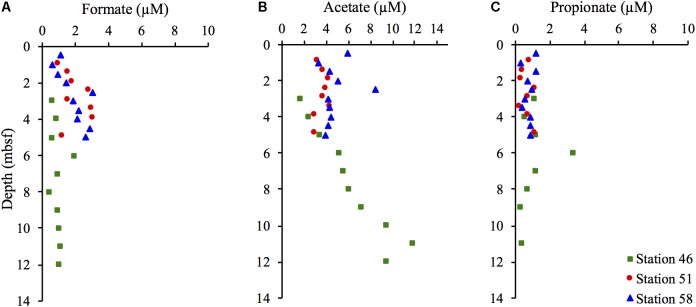
Pore water concentrations of volatile fatty acids (VFAs): formate **(A)**, acetate **(B)**, and propionate **(C)** in μM in samples from the three different stations.

The pore water concentrations of VFA’s were generally low. Formate concentrations at Station 46 remained relatively constant at 1 μM in the zone between 4 and 12 mbsf (**Figure 7A**). At Stations 51 and 58, the formate concentrations showed a slight increase with depth from approximately 1 μM in the uppermost sample to 3 μM at 5 mbsf. At Station 46, the acetate concentration (**Figure 7B**) increased slightly with depth from 1.8 μM at 4 mbsf to 12 μM at 11 mbsf. Propionate concentrations (**Figure 7C**) were similar at all stations and remained in the range of 0.3–1.2 μM with no evident depth trend. The low concentrations are in accordance with previously measured VFA concentrations in marine sediments and are indicative of an active utilization by microorganisms (Glombitza et al., 2015).

## Discussion

### Quality of Buried OM and Controls of Microbial Biomass

In prokaryotic biomass the GlcN:GalN ratio is typically <3.4 (Benner and Kaiser, 2003; Niggemann and Schubert, 2006), while chitinous organisms, such as copepods, have ratios >14 (Benner and Kaiser, 2003). Both, the Gly:Ser and the GlcN:GalN ratios, indicate that the amino acids and amino sugars in the sediment were primarily of prokaryotic origin. This indicates that microorganisms have reworked the amino acids and amino sugars in the original OM from the water column by forming new microbial biomass (Keil and Fogel, 2001) during OM sedimentation in the water column and after its incorporation into the sediment. This observation is consistent with previous studies that have found similar GlcN:GalN ratios (Benner and Kaiser, 2003; Lomstein et al., 2006; Niggemann and Schubert, 2006) and Gly:Ser ratios (Langerhuus et al., 2012), and related these to the bacterial origin of the amino sugars and the amino acids.

**FIGURE 8:**
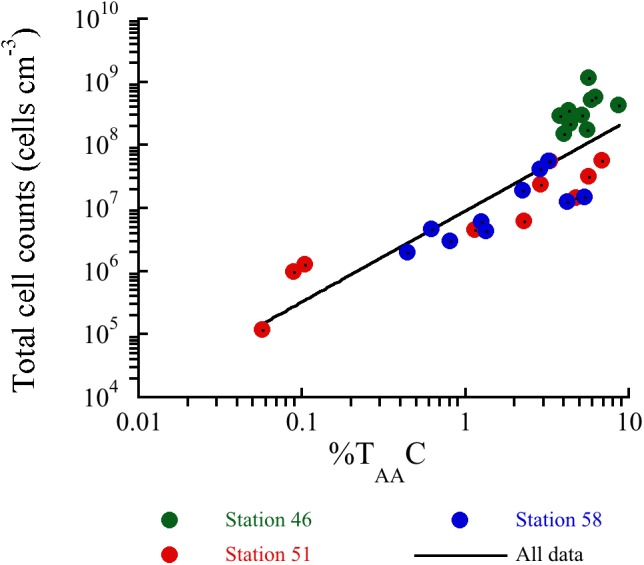
Relationship between total cell counts and the sedimentary %T_AA_C. Regression line shows *y* = 8.94 × 10^6^ × x^(1.44)^, *R* = 0.5614, *N* = 30.

The decrease in %T_AA_C and %T_AA_N (**Figures 3A,B**) at all three stations indicates that the OM became progressively depleted in amino acids with sediment depth. Amino acids are considered as high quality energy sources for microorganisms, since they are degraded faster than bulk OM (Cowie and Hedges, 1992). In previous studies, the %T_AA_C and %T_AA_N have been proven to be useful indicators of the degradation state of sedimentary OM over a broad range of time scales. Lomstein et al. (2012) found %T_AA_C <0.1% in ∼10 million years old sediments off Peru indicating largely degraded OM. The deepest sediment samples at Station 51 and 58 were deposited during the Holocene (<7,000 years ago), but the %T_AA_C and %T_AA_N were similar to values found in ∼10 million years old sediment suggesting that the OM was extensively degraded. In contrast, the sediments at the cold Station 46 show relatively high %T_AA_C- and %T_AA_N-values comparable to %T_AA_C- and %T_AA_N-values found in similar settings (Keil et al., 2000; Braun et al., 2017). Despite their relatively young age, the sediments from the hydrothermal stations are apparently equivalently degraded as the million-year-old sediments off Peru, which indicates that the degree of degradation is not necessarily only a result of sediment age, but also of sediment temperature. The ratios between specific amino acids and their degradation products, Asp:β-Ala and Glu:γ-Aba, further supported the observation that the amino acids became progressively degraded with increasing depth (**Figures 3C,D**) and temperatures. The reactivity of non-protein-bound amino acids is considered to be much lower than the reactivity of protein-bound amino acids (Cowie and Hedges, 1992). This results in decreasing ratios during degradation. Altogether, the amino acid quality indicators at the hydrothermal stations relative to the cold station indicate a strong temperature influence on the degradation of the amino acid pool in sedimentary OM. The strong decrease in OM quality especially at the heated stations means that buried microorganisms need to break down an increasing proportion of lower-quality compounds to gain energy (Braun et al., 2017). Consistent with this, there was a power law relationship between the concentration of vegetative cells and %T_AA_C (**Figure 8**). This indicates that the microbial community size is controlled by the quality of OM and hence the concentration of amino acids and other high quality compounds.

### Indications From Pore Water VFA Concentrations

VFAs (especially acetate) are important intermediates in the microbial degradation and turnover of OM in sediments (Heuer et al., 2009). They are generated by fermentation and hydrolysis of OM and are usually kept at low concentrations in microbially active sediments as a result of their rapid turnover in the terminal processes of OM mineralization, such as sulfate reduction and methanogenesis (Sansone and Martens, 1982; Finke et al., 2007; Glombitza et al., 2015). Analyses of VFAs in the pore water of the three stations showed consistently low VFA-concentrations in the range previously observed in highly active sulfate-reducing marine sediments (Glombitza et al., 2015) – even in the warm, deeper parts of the two hydrothermal stations.

Increases in temperature will potentially increase rates of chemical reactions that release labile substrates from particulate and dissolved OM phases, e.g., hydrolytic release of VFAs. For obvious reasons, this process cannot be sustained indefinitely at high rates without resulting in a significant loss of organic carbon in the sediment. Interestingly, with the exception of the bottom of Station 51, where sediments transition from hemipelagic, OM-rich sediment to metal-sulfide rich, OM-poor hydrothermal sediment, we did not observe a clear decrease in TOC or increase in VFAs with depth at Stations 51 and 58 (**Figures 2A**, **7**), suggesting low rates of OM breakdown even at elevated temperatures. The low VFA concentrations, moreover, indicate that, despite the decrease in microbial population size and the low OM breakdown rates, a tight coupling between VFA generation by fermentation or temperature-driven hydrolysis reactions and VFA consumption by terminal oxidizers is maintained.

### Microbial Turnover of Necromass and Biomass

**FIGURE 9:**
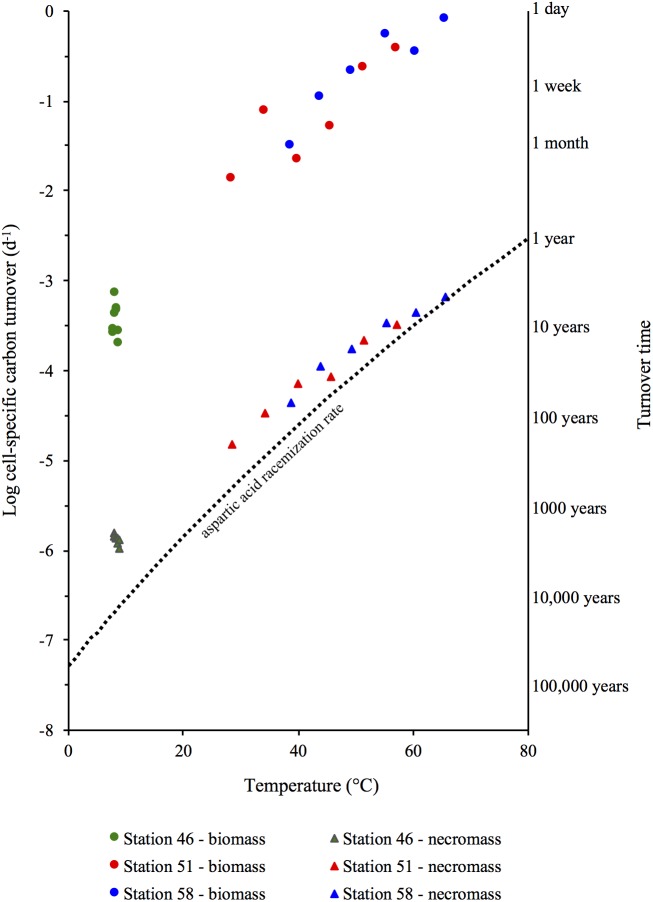
Turnover times of microbial necromass and living microbial biomass in relation to the *in situ* temperature at Station 46, 51, and 58 and the aspartic acid racemization rate constant (*k_i_*-Asp, dotted line).

The D:L-amino acid modeling of microbial necromass and biomass turnover times (**Figure 6**) reveal that the small populations in hot sediments have shorter turnover times (days to months) than the larger populations in the cold sediments (up to tens of years). Microbial turnover times in the order of days to a few months found in the hydrothermal sites are also significantly shorter than the tens to hundreds of years estimated from cold subsurface sediments elsewhere (Braun et al., 2017). Yet, the short microbial turnover times at these sites match the turnover times measured in nutrient rich environments such as soils, lake water, seawater (Jørgensen, 2011), marine surface sediments (<20°C) and in pure cultures of sulfate reducing bacteria (Hoehler and Jørgensen, 2013). The impact of temperature is illustrated by the strong dependence of necro- and biomass turnover time on *in situ* temperature within the temperature interval of 9°C–74°C (*R*^2^ = 0.97 and *R*^2^ = 0.99, respectively, in the log turnover time versus temperature plot, **Figure 9**).

The co-occurrence of short biomass and necromass turnover times and the low quality of the OM, as inferred from the amino acid diagenetic indicators, may at first seem contradictory. It is known that living cells spend a substantial part of the energy gained from substrate metabolism on maintenance, such as repair and replacement of biomolecules, but why should cell-specific biomass turnover rates increase with temperature? The reason may lie in the strong temperature-dependence of biomolecule damaging reactions. As temperature increase, so do rates of biomolecule-damaging reactions, such as amino acid racemization and DNA depurination, and microorganisms are forced to spend more energy on repair and replacement of biomolecules to survive (Lever et al., 2015b). High-temperature adapted microorganisms may reduce these costs, e.g., by having higher GC contents in their DNA, or substituting amino acids with ones that have lower racemization rates at elevated temperature. Presumably, these adaptations provide competitive advantages over microorganisms that lack these adaptations, as greater thermal stability lowers cell-specific energy requirements due to less energy needed for biomolecule repair. We suspect that the strong increase in the fraction of Archaea in the warmer sediment layers of Stations 51 and 58 (**Figure 2**) reflects a shift in community structure toward dominance by high-temperature adapted Archaea. However, these adaptations to temperature do not make thermophilic microorganisms immune to the dramatic increase in biomolecule rates with temperature (e.g., Supplementary Figure A8 in Lever et al., 2015a). Racemization rates of all four amino acids shown in A8 (aspartic acids, glutamic acid, alanin and serin) increase by 2–3 orders of magnitude over the temperature range from surface sediments (9°C) to the bottom of the cores at stations 51 and 58 (65–74°C); over the same temperature range, DNA depurination rates increase by ∼4 orders of magnitude (Supplementary Figure A6 in Lever et al., 2015a), and other biomolecule hydrolysis rates increase by comparable factors (e.g., Wolfenden et al., 1998a,b). Consequently, microorganisms living in surface sediments at Stations 51 and 58 have to cope with at least two orders of magnitude lower amino acid racemization and DNA depurination rates than their counterparts living at the bottom of the cored intervals.

Assuming that energy availability is roughly comparable across the cored intervals of stations 51 and 58, the temperature dependent increase in biomolecule-damaging rates alone can explain why microbial population size at Stations 51 and 58 decreases by approximately two orders of magnitude with depth. Similar apparent temperature-driven decreases in microbial population sizes with depth have been documented elsewhere. In coalbeds that are buried up to 2.5 km offshore Shimokita (Japan), cell numbers drop dramatically at temperatures exceeding 40°C, consistent with a temperature-induced increase in rates of biomolecule damage (Inagaki et al., 2015). Nevertheless, microbial activety and a viable biosphere was identified down to the deepest drilled sediment layers (Inagaki et al., 2015; Glombitza et al., 2016; Liu et al., 2017; Trembath-Reichert et al., 2017).

Our study, thus demonstrates that temperature driven collapses in microbial population size at moderately hot temperatures are not a sole phenomenon of deeply buried sediments. Even “shallow” subsurface sediment layers that are located only a few meters below the seafloor and are cut off from fresh energy inputs show strong temperature-dependent decreases in microbial population size. Yet, the short biomass turnover times indicate that small population sizes in these environments do not necessarily correspond to low microbial activity. Future studies will reveal the contribution of these small, but highly active microbial populations in subsurface sediments at moderately hot temperatures to total microbial activity in sediments and to global biogeochemical cycles in general.

## Author Contributions

MM, BL, ML, and CG designed the study. ML collected the sediment samples and performed the quantification of Bacteria and Archaea. MM did the measurements, analysis and data processing of amino acid composition, amino acid stereochemistry, amino sugars, TOC, and total nitrogen. CG measured VFA concentrations. LD, YM, and FI performed the total cell counts. MD collected and made the interpretation of the temperature data. C-cS and LD determined the sedimentation rates. MM wrote the manuscript. MM, BL, and CG made the outline of the manuscript and data interpretation. All authors reviewed the manuscript.

## Conflict of Interest Statement

The authors declare that the research was conducted in the absence of any commercial or financial relationships that could be construed as a potential conflict of interest.

## Footnotes

1 https://rdp.cme.msu.edu/
